# A network pharmacology approach to evaluate the synergistic effect of dihydromyricetin and myricitrin in vine tea on the proliferation of B16F10 cells

**DOI:** 10.3389/fnut.2022.993133

**Published:** 2022-09-08

**Authors:** Nanxing Zhao, Hongming Kong, Hesheng Liu, Qing Shi, Xiangyang Qi, Qiuping Chen

**Affiliations:** College of Biological and Environmental Sciences, Zhejiang Wanli University, Ningbo, China

**Keywords:** vine tea, dihydromyricetin, myricitrin, proliferation inhibition, network pharmacology, synergistic effect, cell cycle

## Abstract

**Aim of the study:**

Although vine tea has demonstrated broad-spectrum anti-cancer properties, its main active compounds, dihydromyricetin (DMY) and myricitrin (MYT), exert weaker effects than the tea extracts. This study aimed to investigate the synergistic inhibitory effects of DMY and MYT on B16F10 cell proliferation and their synergistic inhibitory effects.

**Methods:**

The effect of vine tea extracts (VTEs) and their active compounds on B16F10 cells was analyzed by 3-(4,5-dimethylthiazol-2-yl)-2,5-diphenyltetrazolium bromide (MTT) assay, fluorescence staining, and flow cytometry. The synergistic effects were calculated by the combination index (CI), and its mechanism was discussed by network pharmacology.

**Results:**

Different VTEs varied in their inhibition of B16F10 cell growth, with IC_50_ values ranging from 4.45 to 12.95 μg/mL, Among these, Guangzhou Qingyuan (Level 2), appeared to have the most potent inhibitory effect. The IC_50_ value of mix-use of DMY and MYT was 19.94∼64.4 μM, of which DMY: MYT = 8:1 had the minimum IC_50_ value of 19.94 μM. Combinations in the 1:1∼8:1 range had stronger effects than the isolated active compound. When they were mixed at the ratio of 1:4∼8:1, CI < 1, showing a synergistic effect. The combination of DMY and MYT also significantly inhibited the tyrosinase activity in B16F10 cells, consistent with its impact on cell proliferation. The eight potential targets were identified by network pharmacology regulating melanin metabolism, tyrosine metabolism, and melanogenesis signaling. According to the analysis of protein-protein interactions, *TP53*, *TNF*, and *TYR* might be critical targets for preventing and treating melanoma.

**Conclusion:**

We found that DMY and MYT induced apoptosis of B16F10 cells, and their combined application had a significant synergistic effect. The present findings indicated that vine tea had a multi-pathway and multi-target impact on the prevention and treatment of melanoma.

## Introduction

Melanoma originates from melanocytes (1, 2). In addition to genetic and other endogenous risk factors, ultraviolet radiation is the most critical exogenous risk factor for melanoma (3). Early detection and surgical resection are the best choices to cure melanoma. At the same time, radiotherapy and chemotherapy are also commonly used treatments (4). However, surgical resection is limited and cannot effectively treat metastatic tumors. Radiotherapy and chemotherapy have drug toxicity and a high cost (5, 6). Therefore, it is important to prevent melanoma through lifestyle. Phytochemicals as supplements have attracted wide attention because of their low cost and toxicity. Many active ingredients have been reported with anti-cancer, anti-metastatic, and pro-apoptotic effects (7–10). Daphnetin inhibits α-MSH-induced melanogenesis *via* PKA and ERK signaling pathways in B16F10 cells and inhibits melanin synthesis in UVB-irradiated HaCaT conditioned medium (11). Paclitaxel in combination with a C-C chemokine receptor type 7 monoclonal antibody can both delay B16F10 cell growth and reduce lymphatic metastasis (12). Luteolin inhibits melanoma growth by regulating cell-cell interaction and oncogenic pathways (13). The purified extract of *Nymphaea hybrid* also has a specific inhibitory effect on melanogenesis in B16F10 cells (14).

Vine tea (*Ampelopsis grossedentata*) has more than 600 years of use in China, is widely distributed in the mountainous areas of southern China, and has been used as a new food resource in recent years. It has hypoglycemic (15), antioxidant (16, 17), antibacterial (18), and anti-inflammatory (19, 20) properties. Several bioactive components have been isolated from vine tea, such as DMY, MYT, and myricetin (21). As the most abundant flavonoid in vine tea, the content of DMY can reach as much as 30% in the leaves (15). Several studies have shown it to be anti-tumor in human lung adenocarcinoma cell lines (22), human glioma (23), and cholangiocarcinoma (24).

Natural products have gained popularity due to their low toxicity and low cost. However, due to their complex composition, the use of natural products is sometimes restricted. Network pharmacology can provide insight into natural products. In network pharmacology, multiple targets of a specific molecule are analyzed through network analysis, emphasizing multi-way regulation of signaling pathways, which can help understand the mechanism of prevention and treatment (25).

The present study evaluated the synergistic effect of the main active components in vine tea, DMY, and MYT, on the proliferation of B16F10 cells. The network pharmacological model was used to explain the underlying mechanisms of vine tea in preventing and treating melanoma.

## Materials and methods

### Materials

B16F10 cells were purchased from Shanghai Institute of Biochemistry and Cell Biology, CAS; MYT standard (CAS: 17912-87-7; ≥ 98%, purity), DMY standard (CAS: 27200-12-0; ≥ 98%, purity), and MTT were purchased from Beijing Solarbio Science and Technology Co., Ltd.; Fetal bovine serum was purchased from Beijing TransGen Biotech Co., Ltd.; RPMI medium and trypsin were purchased from Hyclone from Thermo Fisher Scientific; Hoechst 33342 was purchased from Shanghai Beyotime Biotechnology Co., Ltd.; PI/Rnase staining solution was purchased from Beijing BD Biosciences Co., Ltd., other reagents were commercially available and analytically pure. Six vine tea varieties selected for use in the experiment are all commercially available: A, Wild vine tea in Enshi, Hubei; B, Hubei Enshi selenium-rich vine tea; C, Guangzhou Qingyuan (Level 1); D, Guangzhou Qingyuan (Level 2); E, Wild vine tea in Shiqian, Guizhou; F, Wild vine tea in Zhangjiajie, Hunan.

### Extraction of vine tea

The preparation of vine tea extracts (VTEs) was as follows: 70% ethanol was added to dried vine tea (tea: solvent = 1:10), and the suspension was incubated in a water bath at 40^°^C for 30 min. Then, the extracts were filtered and concentrated in a rotary evaporator to eliminate the solvent. Finally, the concentrate was lyophilized and stored at 4^°^C.

### High-performance liquid chromatography analysis of vine tea extracts from different regions

VTEs were detected using a high-performance liquid chromatography (HPLC) system (Waters, Shanghai, China) with a C_18_ (5 μm, 4.6 × 250 mm) reverse-phase column, and the flow rate was 1.0 mL/min. The mobile phases were 0.1% acetic acid acetonitrile solution (A) and 0.1% acetic acid aqueous solution (B). Under the following gradient profile: 0–22 min 10–30% A, followed by washing and reconditioning the column (3 min). The detection wavelength was 254 nm (200–400 nm full-band scanning).

The standard curve equation of DMY was *y* = 9656905.4141x+39250.3632, *r*^2^ = 0.9995 (0.1677 ∼ 0.8 mg/mL); and MYT was *y* = 33785171.6504x+446.4681, *r*^2^ = 0.9998 (0.0030 ∼ 0.035 mg/mL). The VTEs were prepared at a 1 mg/mL concentration, the sample injection volume was 20 μL and repeated three times as parallels.

### Cell culture

B16F10 cells were inoculated in RPIM-1640 medium containing 10% FBS at 37^°^C and cultured in an incubator with 5% CO_2_ and saturated humidity (MCO-15AC, Sanyo, Japan), and the medium was changed every other day. When cells reached about 80% confluence, they were digested and subcultured with 0.25% trypsin and allowed to continue to develop (26).

### The proliferation of B16F10 cells by MTT assay

B16F10 cell suspension was inoculated in a 96-well plate at 3,000 cells/well for 24 h. VTEs were dissolved in dimethyl sulfoxide (DMSO) to 100 mg/mL and diluted with a culture medium to 10–30 μg/mL. For the administration of DMY and MYT, the compounds were diluted in DMSO to 100 mM and then diluted with a culture medium to get the needed concentration. The final concentration of the DMSO was less than 0.5% in the medium.

To evaluate the synergistic effect of DMY and MYT, cells were treated with DMY, MYT, or mix-use. In the mixture, the compounds were present in ratios of 1:4, 1:2, 1:1, 2:1, 4:1, and 8:1. After treatment for 72 h, 10 μL of 5 mg/mL MTT was added to each well. The media containing MTT was removed after 4 h, and 100 μL DMSO was added to each well to dissolve the formazan crystals (27). The plate was shaken for 1 min, the absorbance at 490 nm was measured, the inhibition rate of the drug on cell growth was calculated, and the general equation for the dose-effect relationship was obtained according to the following formula. The combination index (CI) was calculated after 72 h incubation, and the CI value represented the combined effect of two drugs; CI <1 showed synergism, CI = 1 additive effects, and CI > 1 showed antagonism. The CI values of different intervals had strong and weak differences. CI value was calculated by the equation.


$$log(fa/fu)=log{\left(D/D_{m}\right)}^{m}=mlog(D)-mlog(D_{m})$$

D: the dose of the drug

D_*m*_: the median-effect amount is signifying the potency.

Fa: the fraction affected by the dose

fu: the fraction unaffected, fu = 1-fa

If b = m, a = -mlogD_*m*_, Y = log (fa/fu), X = logD, Y = bX+a.


$$\text{CI}=\frac{{\left(D\right)}_{1}}{{\left({\text{D}}_{X}\right)}_{1}}+\frac{{\left(D\right)}_{2}}{{\left({\text{D}}_{X}\right)}_{2}}$$

In the above formula, (D)_1_ and (D)_2_ represent the combined inhibition rate X% of drug 1 and drug 2 in the experiment, and (D_*X*_)_1_ and (D_*X*_)_2_ represent their respective inhibition rates X% (28).

### Cell fluorescence staining

B16F10 cells were inoculated in 24-well plates at 3 × 10^4^ cells/well. DMY, MYT, or mixed-use were applied the next day at a concentration of 75 μM. The cells were stained for 72 h. The original medium was aspirated and discarded, and the well was washed with PBS. Each was stained with 250 μL Hoechst 33342 solution. After 15 min, the dye solution was discarded. After washing 3 times with PBS, fluorescence photomicrographs were obtained by an inverted fluorescence microscope (IX73, Olympus, Japan) (29) and quantitatively analyzed using ImageJ software.


Mean=IntDen/Area(30).

Mean: Mean gray value.

IntDen: Integrated Density.

### Effects of dihydromyricetin and myricitrin on tyrosinase activity in B16F10 cells

B16F10 cells were inoculated into 96-wells at 3,000 cells/well. The cells were cultured for 24 h before administering the test compounds. DMY and MYT were in DMSO to 100 mM and diluted with a culture medium to a concentration gradient of 20–100 μM, either alone or in combination.

After B16F10 cells were treated for 72 h, 90 μL 1% TritonX-100 was added to each well, and then 10 μL 1.0 mg/mL L-DOPA was added. The absorbance at 490 nm was measured after 5 min of ultrasound and treatment at 30^°^C for 30 min. The enzyme activity was calculated using the following formula: tyrosinase activity = OD *_sample_* /OD*_control_* × 100% (31).

### Determination of cell cycle

The digested B16F10 cells were inoculated into three 12-well plates as three parallel groups at 5 × 10^4^ cells/well. After 24 h, the cells were adherent to the wall before treatment. 75 μM DMY, MYT, or the combined compounds were added to each well, and then the cells were incubated for 24 h. The digested cells were collected and centrifuged at 1,000 rpm for 5 min; then, the supernatant was discarded. After washing the cells with PBS twice, 4^°^C 75% ethanol was added slowly, and the cells were kept in darkness at 4°C overnight. The cells were centrifuged at 1,000 rpm for 5 min before analysis, the supernatant was discarded, and the cells were washed with PBS to remove all ethanol and resuspended in 0.5 mL PI/Rnase staining solution, incubated in the dark at room temperature for 15 min, and then analyzed by flow cytometry (FACSVerse, BD, America) (32).

### Network analysis of component-disease interactions

The genes of targets associated with “melanoma” disease names were collected through CTD,^1^ and the genes of targets with “DMY” and “MYT” as chemical names were similarly searched. Target genes were screened for overlapping with DMY, MYT, and melanoma. Then, they were uploaded to Cytoscape 3.8.2 to generate a network map of component-gene-disease interactions, and the protein interaction diagrams, gene ontology (GO), and kyoto encyclopedia of genes and genomes (KEGG) pathway diagrams were obtained for further analysis.

### Data analysis and processing

Calcusyn 2.0 software was used to calculate the CI of DMY and MYT for further analysis. The data were visualized using by Origin 9.0 and were statistically analyzed by SPSS. For statistical analysis, one-way ANOVA was used. Results are presented as means and error bars represent standard deviation (SD). *p* < 0.05 was considered statistically significant.

## Results

### High-performance liquid chromatography analysis of vine tea extracts

**FIGURE 1:**
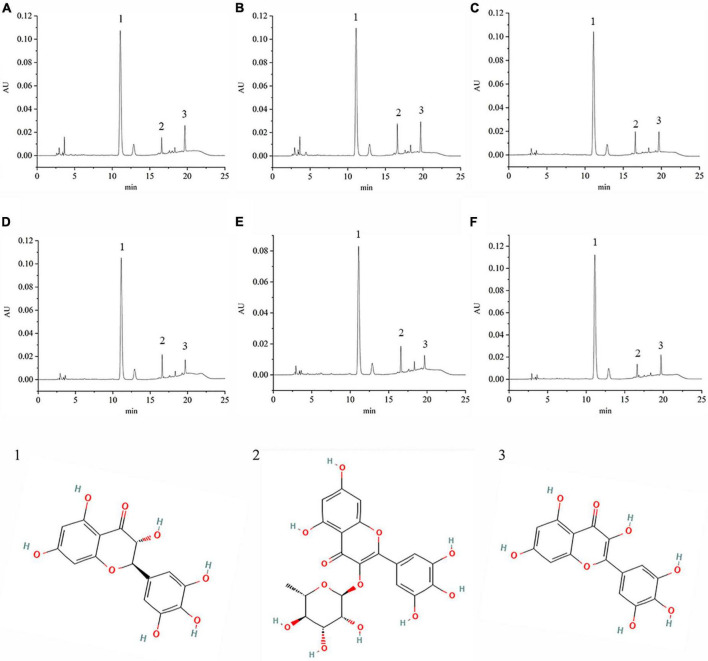
The chromatography of different vine teas. **(A)** Wild vine tea in Enshi, Hubei. **(B)** Hubei Enshi selenium-rich vine tea. **(C)** Guangzhou Qingyuan (Level 1). **(D)** Guangzhou Qingyuan (Level 2). **(E)** Wild vine tea in Shiqian, Guizhou. **(F)** Wild vine tea in Zhangjiajie, Hunan; 1, Dihydromyricetin; 2, Myricitrin; 3, Myricetin.

**FIGURE 2:**
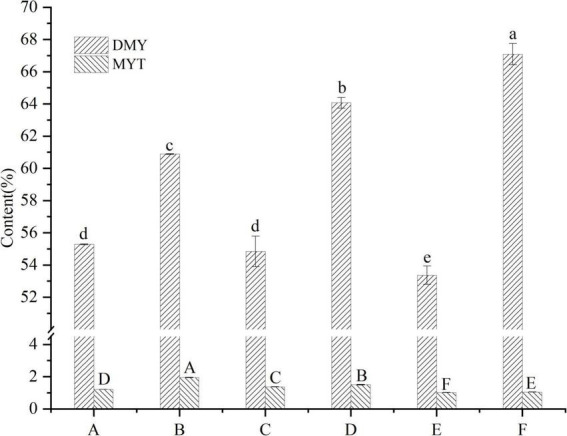
Contents of DMY and MYT in the extract of different vine teas. Values are means ± SD (*n* = 3). Different groups of vine tea were prepared at 1 mg/mL, DMY and MYT standards were prepared at different concentrations with methanol solution and were determined by HPLC. Different letters and capitalization indicate significant differences between means at *P* < 0.05 by one-way ANOVA followed by Duncan comparison test. DMY, dihydromyricetin; MYT, myricitrin.

VTEs were analyzed by HPLC (Figure 1), and three peaks revealed the main components of vine tea were DMY, MYT, and myricetin. The range of DMY was 53.36 ∼ 67.09%, of which the wild vine tea in Zhangjiajie, Hunan province, was the highest, with a content of 67.09% (Figure 2).

### Inhibitory effects of vine tea extracts on the proliferation of B16F10 cells

**FIGURE 3:**
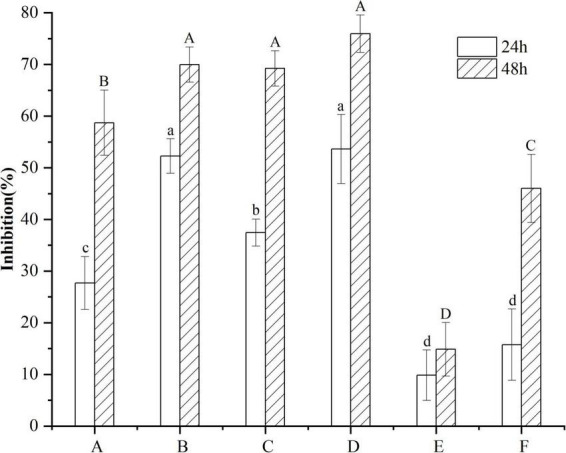
Inhibitory effect of different kinds of vine tea extracts on the proliferation of B16F10 cells. Values are means ± SD (*n* = 3). B16F10 cells were treated for 24 h with inhibitor (10 μg/mL VTEs of different varieties) and medium (control), and cell viability was determined using the MTT assay. Different letters and capitalization indicate significant differences between means at *P* < 0.05 by one-way ANOVA followed by Duncan comparison test. (A) Wild vine tea in Enshi, Hubei. (B) Hubei Enshi selenium-rich vine tea. (C) Guangzhou Qingyuan (Level 1). (D) Guangzhou Qingyuan (Level 2). (E) Wild vine tea in Shiqian, Guizhou. (F) Wild vine tea in Zhangjiajie, Hunan.

B16F10 cells were treated with 10 μg/mL VTEs, and the inhibition rate of cell proliferation increased gradually within 48 h (Figure 3). Within 24 h, the inhibition rate of VTEs on B16F10 cells was 9.87 ∼ 53.62%. The inhibition rate was 14.87 ∼ 75.95% within 48 h; the results showed that vine tea had an inhibitory effect on B16F10 cells. Among these, sample D had the best inhibitory effect.

### Correlation analysis

As shown in Table 1, Quantitative Composition-Activity Relationship analysis showed a very significant correlation between DMY and the proliferation inhibition rate of B16F10 cells, which indicated that DMY in vine tea played an essential role in inhibiting the proliferation of B16F10 cells.

**TABLE 1:** Correlation analysis.

Administration time	24 h		48 h	
Single use	R	*P*	R	*P*
DMY	–0.882	<0.001	0.713	0.009
MYT	0.443	0.150	0.423	0.170
Myricetin	–0.562	0.057	0.540	0.070

Previous experiments showed that the IC_50_ value of DMY on B16F10 cells was 14.73 μg/mL. IC_50_ of VTEs on B16F10 cells was 4.45∼12.95 μg/mL, in which sample D showed the best inhibitory effect (Figure 3). The IC_50_ values of six VTEs were lower than DMY, indicating that VTEs had a better inhibitory effect on B16F10 cells. Therefore, we inferred that MYT in vine tea had no significant correlation with inhibiting B16F10 cell proliferation, but MYT may have a synergistic effect.

### Inhibition of B16F10 cell proliferation by dihydromyricetin and myricitrin

**FIGURE 4:**
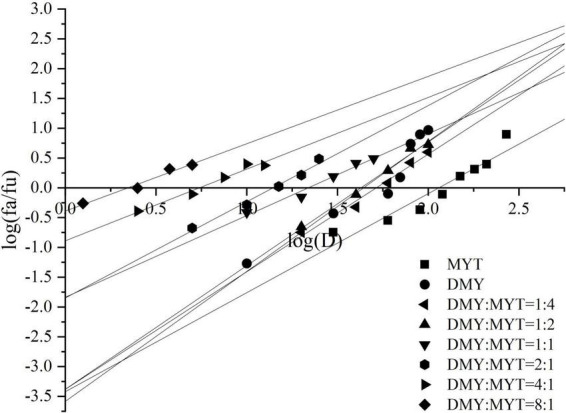
The effects of DMY and MYT on B16F10 cell proliferation. Values are means ± SD (*n* = 3). B16F10 cells were treated with DMY, MYT, or mix-use (1:4 ∼ 8:1) for 72 h, before assessment of viability by MTT assay. The dose-response relationship was obtained by transformation according to the equation. DMY, dihydromyricetin; MYT, myricitrin.

**FIGURE 5:**
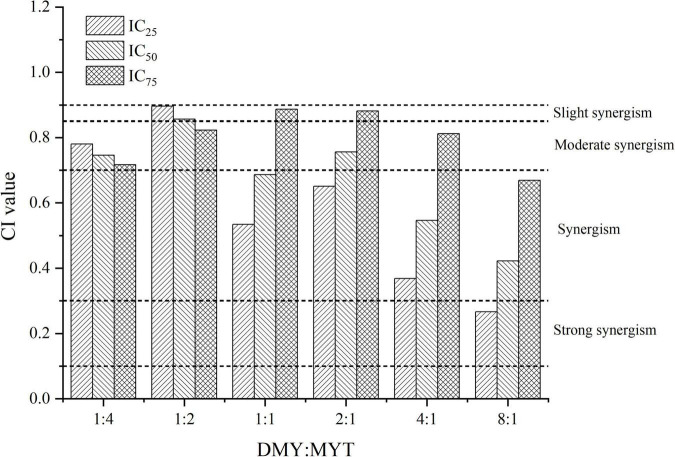
Analysis of the combined inhibitory effects of DMY and MYT on B16F10 cell proliferation. Values are means ± SD (*n* = 3). B16F10 cells were treated with DMY, MYT, or mix-use (1:4 ∼ 8:1) for 72 h, before assessment of viability by MTT assay. The CI value was calculated by the equation. DMY, dihydromyricetin; MYT, myricitrin.

Single or combined administration of DMY and MYT could inhibit the proliferation of B16F10 cells. The rate of cell proliferation inhibition increased gradually in a dose-dependent manner as drug concentration increased (Figure 4). When the content of DMY in the mixed drug increased, the IC_50_ decreased gradually. When the DMY: MYT was more significant than 1: 1, the IC_50_ of the mix-use group was lower than that of the single-drug group, and the lowest IC_50_ was 19.94 μM when DMY: MYT = 8:1 (Table 2). The CI value was used to analyze the experimental results further. DMY and MYT had a synergistic effect on inhibiting the proliferation of B16F10 cells. When they were mixed in the ratio of 1:4–8:1, the CI at IC_25_, IC_50_, and IC_75_ was less than 1, showing a synergistic effect. Among them, the CI of IC_25_ was the lowest, and DMY: MYT = 8:1 showed strong synergism, indicating that the synergistic effect of DMY and MYT was more apparent when the cell inhibition rate was low. Among different proportions, when the ratio of them was 8:1, the CI value was the lowest, indicating that the synergistic effect of the ratio was the best (Figure 5).

**TABLE 2:** Half-inhibitory concentration of drugs on B16F10 cell proliferation.

Sample	DMY	MYT	DMY: MYT					
			1:4	1:2	1:1	2:1	4:1	8:1
IC_50_ (μM)	45.98	109.1	64.64	63.89	43.65	41.87	27.41	19.94
r	0.9	0.95	0.99	0.99	0.99	0.99	0.98	0.98

### Effects of combination of dihydromyricetin and myricitrin on tyrosinase activity in B16F10 cells

**FIGURE 6:**
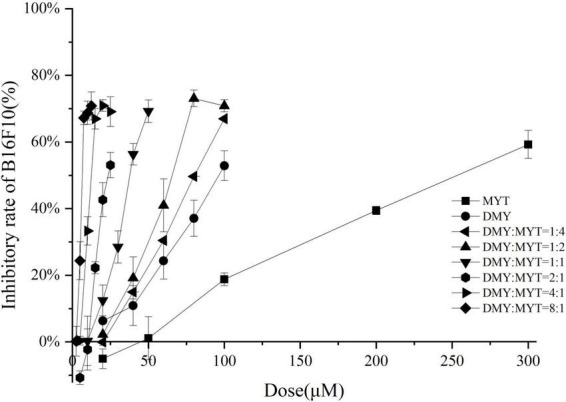
Tyrosinase inhibition by DMY and MYT in B16F10 cells. Values are means ± SD (*n* = 3). After B16F10 cells were treated with DMY, MYT, or mix-use (1:4 ∼ 8:1) for 72 h, 90 μL of 1% TritonX-100 was added to each well, and then 10 μL of 1.0 mg/mL L-DOPA was added. The absorbance at 490 nm was measured after 5 min of ultrasound and treatment at 30^°^C for 30 min. The dose-response relationship was obtained by transformation according to the equation. DMY, dihydromyricetin; MYT, myricitrin.

**FIGURE 7:**
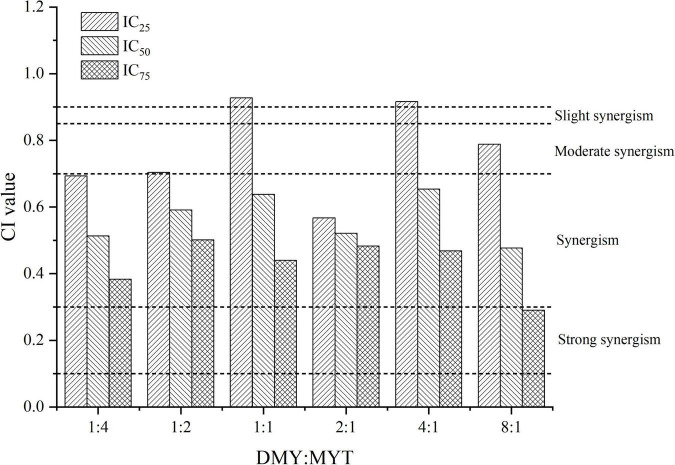
Analysis of the combined effect of DMY and MYT on inhibiting tyrosinase in B16F10 cells. Values are means ± SD (*n* = 3). After B16F10 cells were treated with DMY, MYT, or mix-use (1:4 ∼ 8:1) for 72 h, 90 μL of 1% TritonX-100 was added to each well, and then 10 μL of 1.0 mg/mL L-DOPA was added. The absorbance at 490 nm was measured after 5 min of ultrasound and treatment at 30^°^C for 30 min. The dose-response relationship was obtained by transformation according to the equation. The CI value was calculated by the equation. DMY, dihydromyricetin; MYT, myricitrin.

DMY and MYT inhibited tyrosinase activity in B16F10 cells when applied alone or in combination. With the increase in drug concentration, the inhibition of tyrosinase activity increased gradually, showing a dose-dependent relationship in which the activity of DMY was more substantial than MYT’s (Figure 6). The IC_50_ of the combined drug group was lower than that of the single-drug group. The lowest IC_50_ of DMY: MYT = 2:1 was 62.59 μM (Table 3). When they were mixed in the ratio of 1:4–8:1, the CI of IC_25_, IC_50_, and IC_75_ was less than 1, showing a synergistic effect. Among them, the CI of IC_75_ was the smallest, and DMY: MYT = 8:1 showed strong synergism, indicating that the synergistic effect was higher when DMY and MYT were used together. Among the different ratio concentrations, the CI value of DMY: MYT = 8:1 was the lowest at IC_50_ and IC_75_, while the lowest CI at IC_25_ was the ratio of 2:1 (Figure 7).

**TABLE 3:** Half-inhibitory concentrations of drugs on tyrosinase in B16F10 cells.

Sample	DMY	MYT	DMY: MYT					
			1:4	1:2	1:1	2:1	4:1	8:1
IC_50_ (μM)	107.7	233.95	98.68	99.42	75.67	62.59	78.91	73.07
r	0.97	0.97	1	0.99	0.99	0.97	0.93	0.94

### Inhibition of B16F10 cell proliferation by dihydromyricetin and myricitrin—Fluorescence staining

**FIGURE 8:**
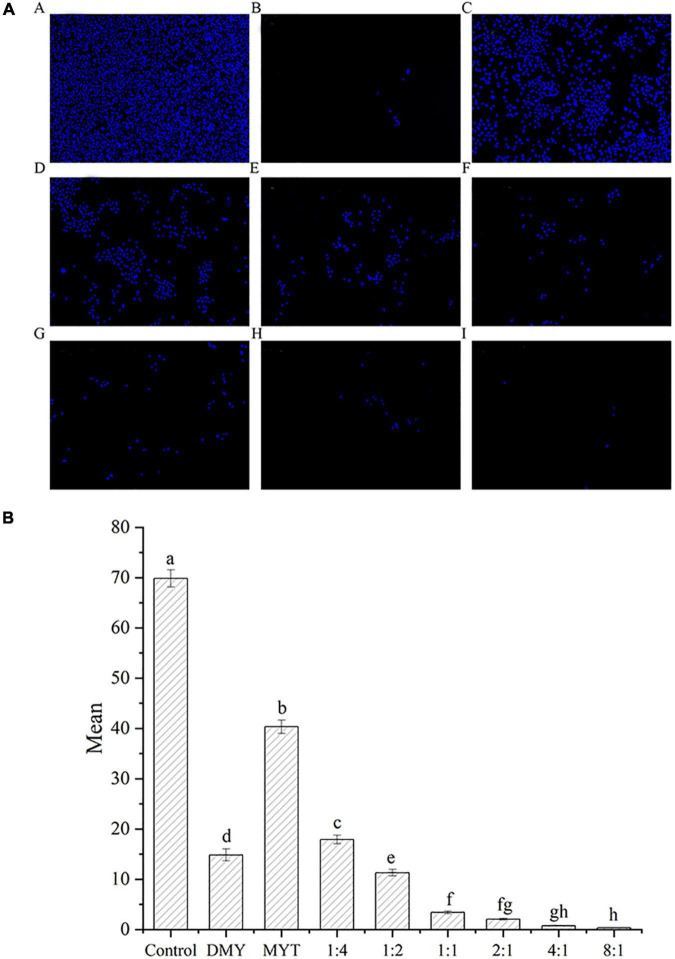
Analysis of the synergistic inhibitory effect of DMY and MYT on cell proliferation by fluorescence staining. Cell viability was determined by Hoechst 33342 fluorescent staining and pictures were quantified with ImageJ. Values are means ± SD (*n* = 3). Different letters indicate significant differences between means at *P* < 0.05 by one-way ANOVA followed by Duncan comparison test. **(A)** Fluorescence photography. **(B)** Fluorescence quantitative analysis. (A) Control. (B) DMY. (C) MYT. (D) DMY: MYT = 1:4. (E) DMY: MYT = 1:2. (F) DMY: MYT = 1:1. (G) DMY: MYT = 2:1. (H) DMY: MYT = 4:1. (I) DMY: MYT = 8:1. DMY, dihydromyricetin; MYT, myricitrin; Mean, mean gray value.

Hoechst’s staining results showed that DMY and MYT alone or in combination could effectively inhibit the proliferation of B16F10 cells and promote cell apoptosis compared with a control group. The fluorescent staining results were consistent with the MTT experimental data. With the increase of DMY concentration in the combined drug group, the number of cells decreased gradually, in which the single DMY was stronger than MYT. The best effect was at 8:1 in the combined drug group (Figure 8A). The quantitative fluorescence results showed that the 8:1 group had the lowest mean fluorescence intensity, which was consistent with the image results (Figure 8B).

### Effects of B16F10 cells on cell cycle in combination with dihydromyricetin and myricitrin

The effects of DMY and MTY on the cell cycle of B16F10 cells were analyzed by flow cytometry. As shown in Table 4, the results of DMY, MYT, and the combination group on the cell cycle of B16F10 cells were mainly characterized by reducing the number of cells passing through G1 phase and blocking the cell cycle in S and G2 phases. The cell cycle results of the combination group (1:4, 1:2, 1:1, 2:1, 4:1, and 8:1) were similar to those of the DMY group, suggesting that their mechanisms of action were relatively consistent. Compared with the control group, the experimental groups had significant differences, of which DMY: MYT = 8:1 group was the most prominent, and the percentage of cells in the G1 phase decreased to 59.38%, the rate of cells in the S phase increased to 33.16% and the cells in G2 phase increased to 7.46%.

**TABLE 4:** Effects of the drugs on the cell cycle of B16F10 cells.

Sample	Control	DMY	MYT	DMY: MYT					
				1:4	1:2	1:1	2:1	4:1	8:1
G1/%	75.9 ± 0.58a	59.11 ± 2.00d	67.81 ± 5.3b	63.5 ± 0.75c	59.32 ± 0.05d	60.36 ± 1.39cd	62.17 ± 1.26cd	59.96 ± 0.45cd	59.38 ± 1.43cd
S/%	22.05 ± 1.39d	35.32 ± 1.10a	27.25 ± 4.97c	28.51 ± 0.75bc	35.73 ± 0.13a	33.56 ± 1.41ab	32.44 ± 1.53ab	34.79 ± 1.01a	33.16 ± 1.2ab
G2/%	2.05 ± 0.82d	5.57 ± 0.94bc	4.96 ± 0.33c	7.98 ± 0.03a	4.96 ± 0.17c	6.08 ± 0.39b	5.39 ± 0.29bc	5.26 ± 0.57bc	7.46 ± 0.23a

The mean value of different letters in the same row is significantly different from the mean value of control (*P* < 0.05).

### Targets of dihydromyricetin and myricitrin on melanoma

**FIGURE 9:**
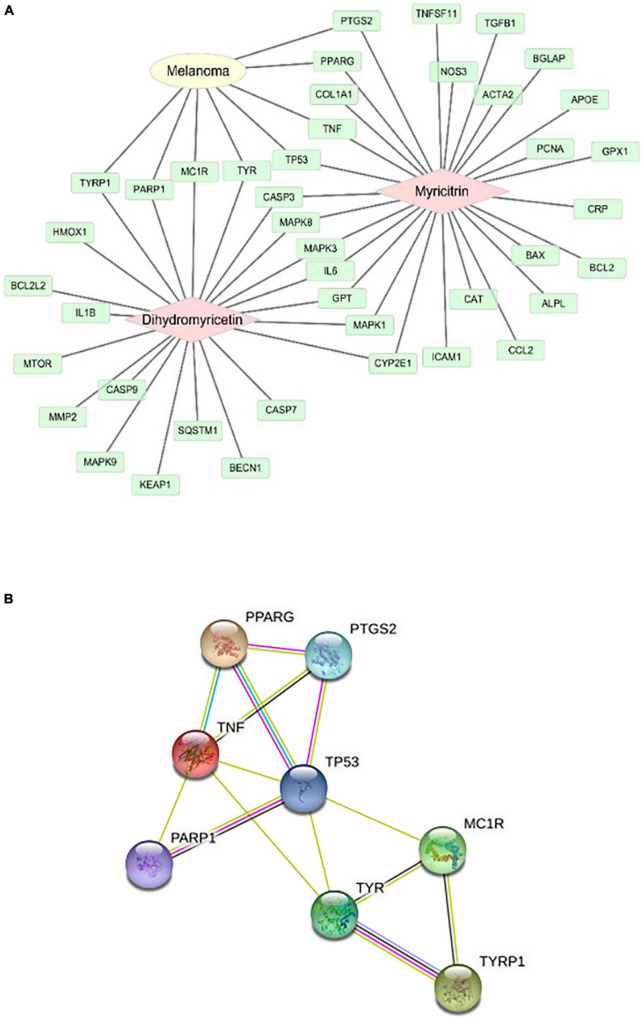
Analysis of action targets of DMY and MYT on melanoma. **(A)** The targets of dihydromyricetin and myricitrin on melanoma. **(B)** The protein-protein interaction network of DMY, MYT, and melanoma disease.

The mechanism of action of vine tea in the prevention and treatment of melanoma was studied. The targets of DMY and MYT on melanoma were shown in Figure 9A. 8 targets of DMY and MYT related to melanoma, among which DMY acted on *TYRP1*, *PARP1*, *MC1R*, and *TYR*, while MYT acted on *TP53*, *TNF*, *PPARG*, and *PTGS2*.

Two components-disease cross targets were uploaded to the String database to construct a protein-protein interaction network (PPI). In this network, eight targets could interact with proteins, and 14 edges represent the interactions between proteins. The average degree of freedom of each node in the network was 3.5, the average betweenness centrality was 0.101190, and the average closeness centrality was 0.645117 (Figure 9B). There were three targets above the average, speculating that *TP53*, *TNF*, and *TYR* might be the critical targets of vine tea in preventing and treating melanoma (Table 5). Interleukin-4 and 13 signaling, thyroid cancer, pathways in cancer, signaling by interleukin, interleukin-10 signaling, melanogenesis, melanin biosynthesis, and tyrosine metabolism might be the key to treating melanoma.

**TABLE 5:** Basic information of crucial gene in the control of melanoma by vine tea.

Gene	Pathway	Degree	Betweenness	Closeness
*TP53*	Interleukin-4 and 13 signaling, thyroid cancer, pathways in cancer, signaling by Interleukins	6	0.381	0.875
*TNF*	Interleukin-4 and 13 signaling, signaling by interleukins, interleukin-10 signaling	5	0.167	0.778
*TYR*	Melanogenesis, melanin biosynthesis, tyrosine metabolism	4	0.190	0.700

### GO pathway enrichment analysis

**FIGURE 10:**
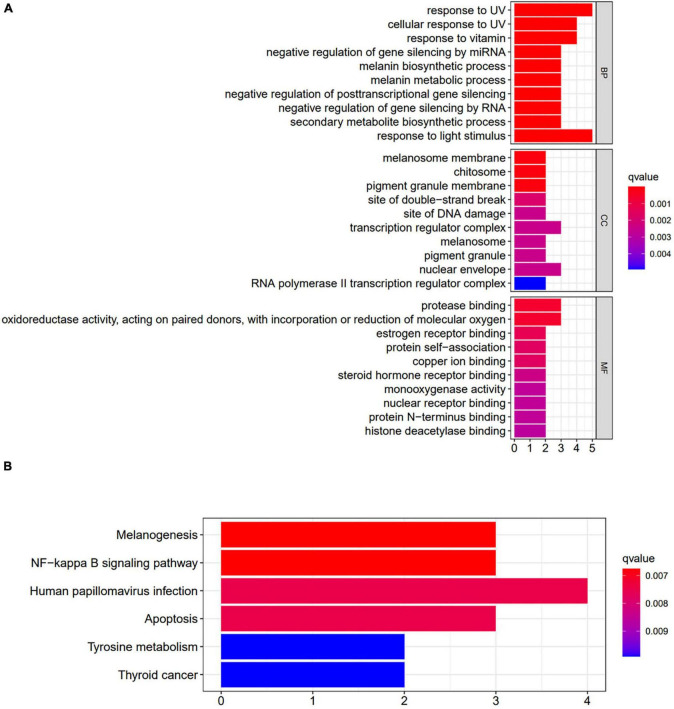
Enrichment analysis of key genes. **(A)** Results of GO enrichment analysis. **(B)** Results of KEGG enrichment analysis. BP, biological process; CC, cellular component; MF, molecular function.

The functional enrichment analysis of GO terms was carried out. A total of 474 enrichment results were obtained in the biological process (*P* < 0.01), and 10 categories with the highest functional values were selected, mainly related to the metabolic synthesis of melanin, including the corresponding response to light stimulation and the negative regulation of gene silencing. Twenty six enrichment results were obtained by molecular function, mainly related to oxidoreductase activity, and 12 enrichment results were obtained by cell composition, including melanosome membrane, and complex transcription mechanism (Figure 10A).

### KEGG pathway enrichment analysis

The selected targets were analyzed by KEGG pathway enrichment analysis, and 6 enrichment results were obtained (*P* < 0.01). It included the melanogenesis signaling pathway, NF-κB signaling pathway, tyrosine metabolism signaling pathway, thyroid cancer signaling pathway, and apoptosis signaling pathway, suggesting that DMY and MYT play a role in preventing and treating melanoma by acting on the multiple pathways (Figure 10B).

## Discussion

Melanoma is challenging to treat because of its ability to metastasize at early stages and its resistance to conventional cancer treatments (33, 34). The use of molecular targeted drugs and immunotherapy for melanoma is limited by the high cost and significant side effects (2). Therefore, safer and more effective treatments are necessary. Natural products have few side effects and do not contain any drug residues. The compatibility of active components can improve disease prevention and treatment efficacy and has been widely used in anti-tumor. For example, *Cuphea aequipetala* extracts can induce cell accumulation in the G1 phase of the cell cycle, induce apoptosis, and thus exhibit inhibitory activity (35). Menke et al. reported that dandelion extract and mistletoe extract could promote neuroblastoma cell apoptosis (36). Sturza et al. found that quercetin could simultaneously regulate the pathway of glycolysis and mitochondria to produce ATP to kill cancer cells (37). Some studies have shown that flavonoids can inhibit *mTOR* and *RAS* carcinogenic pathways, activate apoptosis, and lead to cell cycle stagnation. The vine tea contains many flavonoids, which have certain biological activities in cells and have anti-tumor effects (38). Huang et al. found that DMY inhibits melanin synthesis through its antioxidant properties and down-regulation of protein kinase A, protein kinase C, and mitogen-activated protein kinase signal pathways (39). Our data showed that the VTEs could inhibit B16F10 cells, and the inhibitory effect on tyrosinase was consistent with cell inhibition experiments. In organisms, tyrosinase is the key enzyme involved in melanin biosynthesis (31). It has been reported that individual flavonoids are potential melanin synthesis inhibitors in mammalian melanocytes (40, 41). For melanoma diseases, only flavonoids with an IC_50_ value of less than 50 μM can inhibit mammalian tyrosinase, thus reducing the melanin synthesis of B16F10 (31).

Some studies have shown that natural products target pathogens through a combination of different structures and functions (42). The active components in vine tea are present as mixtures. The activity of purified extracts of single compounds is weaker than that of crude extracts, suggesting that maximum bioactivity is obtained through the interaction of different functional factors (43). Previous studies have compared the theoretical value (T-EM) with the actual experimental value (EM). If the EM is higher than the T-EM, the two compounds are considered to have a synergistic effect. The EM value is the sum of the effect values of each combination. For example, it has been found that the synergistic effect of EGCG and metformin can increase ROS, thereby destroying the ribonucleic acid of B16F10 cells and promoting cell apoptosis (44). These flavonoids have also been shown to inhibit melanoma (37). This inhibitory effect has two main aspects: inhibiting cancer cell proliferation and promoting cancer cell apoptosis (45–47). Recent studies have shown inhibitory effects on the expansion of human acute promyelocytic leukemia cells and K562 cells (48), Bel-7402 cells (49), human breast cancer cells (50, 51), and nasopharyngeal carcinoma HK –1 cells (27). We expected that both DMY and MYT would have inhibitory effects on B16F10 cells. However, the IC_50_ value of DMY on B16F10 cells was 14.73 μg/mL, the IC_50_ of MYT on B16F10 cells was 50.66 μg/mL, and the IC_50_ of VTEs on B16F10 cells was 4.45∼12.95 μg/mL, which showed that the effect was not as significant as a crude extract. We hypothesized that the combination of active compounds in the extracts could enhance the inhibitory effect on melanoma disease. Therefore, the correlation between DMY, MYT, and myricetin in vine tea was analyzed, and DMY had a very significant correlation. Further inhibition experiments showed that the IC_50_ of VTEs was lower than that of DMY. There might be a synergistic effect between the active compounds. We selected DMY and MYT for further study since our previous experimental results indicated that DMY and myricetin had no synergistic effect, whereas DMY and MYT did show an effect. B16F10 cells were treated with different ratios of DMY and MYT, ranging from 1:4 to 8:1; it was found that DMY: MYT = 8:1 was the most effective in inhibiting B16F10 and tyrosinase activity. Fluorescence staining showed that the number of cells decreased with the increase of DMY concentration, and the inhibitory effect of DMY was more substantial than MYT’s. Compared with the control group, the proportion of cells in S phase, or G2 phase was increased, but G1 phase was decreased. The effects of DMY and MTY on B16F10 cells were analyzed by flow cytometry, which showed that the cell cycle was arrested in the S phase and G2 phase. The combined treatment could effectively reduce the number of cells entering the G1 phase. The inhibition of DMY on melanoma cells was higher than that of MYT. We speculated that the proportion of DMY had an important effect on the synergism. It was observed that the IC_50_ values of the mix-drugs decreased with increased DMY content. Especially in the range of 1:1∼8:1, they showed lower IC_50_ than single-use. Based on CI and isobole methods, the 8:1 group showed a strong synergistic effect. However, only a narrow range of ratios were tested in this experiment; perhaps increasing the ratios will produce stronger effects, which will be tested in the follow-up experiments.

Furthermore, network pharmacology was used to create a network map of the “active ingredient-acting gene-disease” of vine tea on melanoma disease. It was found that DMY and MYT have the same target and act on eight different targets of melanoma disease, which might be the reason for the synergism between DMY and MYT. Based on the interaction analysis of these targets, *TP53, TNF, and TYR* might be the key targets for the prevention and treatment of melanoma diseases. P53 protein promoted cancer cell apoptosis by responding to the anti-proliferation effects of various physiological processes such as aging (52). GO, and KEGG enrichment analysis found that the prevention and treatment of melanoma were related to ultraviolet reaction, melanosome membrane, chitosan, receptor binding, NF-κB signal pathway, and apoptosis. Abnormal expression of NF-κB has been associated with various cancers (53). Exposure to ultraviolet (UV) radiation, that is, UVA (315–400 nm), and UVB (280–315 nm), is considered to be a significant risk factor for melanoma (54). Melanosomes could synthesize and organize melanin. Melanin synthesis and melanosome transport disorders are associated with pigmented diseases (55). The crosstalk between protease-activated receptor 1 and platelet-activating factor receptor has been demonstrated to regulate the expression of melanoma cell adhesion molecule (MCAM/MUC18) metastasis of melanoma (56).

## Conclusion

The combination of DMY and MYT in vine tea could synergistically inhibit the proliferation of B16F10 cells, and they have a synergistic effect on different targets. Through network pharmacology, it is concluded that *TP53*, *TNF*, and *TYR* are the main targets of DMY and MYT in melanoma disease and regulate signaling pathways such as melanogenesis, NF-κB, and apoptosis. Regular application of low-toxicity and vine tea extract can contribute to the prevention and treatment of melanoma.

## Data availability statement

The original contributions presented in this study are included in the article/supplementary material, further inquiries can be directed to the corresponding author/s.

## Author contributions

QC and XQ designed the study. NZ and HK performed the experiments, sorted out the data, and drew the drawings. HL and QS provided the experimental guidance. NZ participated in manuscript writing. QC revised the manuscript. All authors contributed to the article and approved the submitted version.

## Footnotes

1
http://ctdbase.org/
